# Event-Related Potential Correlates of Performance-Monitoring in a Lateralized Time-Estimation Task

**DOI:** 10.1371/journal.pone.0025591

**Published:** 2011-10-19

**Authors:** Theo O. J. Gruendler, Markus Ullsperger, René J. Huster

**Affiliations:** 1 Cognitive Neurology, Max Planck Institute for Neurological Research, Cologne, Germany; 2 Biological Psychology, Radboud University Nijmegen, Nijmegen, Netherlands; 3 Experimental Psychology Lab, Institute for Psychology, University of Oldenburg, Oldenburg, Germany; University of Granada, Spain

## Abstract

Performance-monitoring as a key function of cognitive control covers a wide range of diverse processes to enable goal directed behavior and to avoid maladjustments. Several event-related brain potentials (ERP) are associated with performance-monitoring, but their conceptual background differs. For example, the feedback-related negativity (FRN) is associated with unexpected performance feedback and might serve as a teaching signal for adaptational processes, whereas the error-related negativity (ERN) is associated with error commission and subsequent behavioral adaptation. The N2 is visible in the EEG when the participant successfully inhibits a response following a cue and thereby adapts to a given stop-signal. Here, we present an innovative paradigm to concurrently study these different performance-monitoring-related ERPs. In 24 participants a tactile time-estimation task interspersed with infrequent stop-signal trials reliably elicited all three ERPs. Sensory input and motor output were completely lateralized, in order to estimate any hemispheric processing preferences for the different aspects of performance monitoring associated with these ERPs. In accordance with the literature our data suggest augmented inhibitory capabilities in the right hemisphere given that stop-trial performance was significantly better with left- as compared to right-hand stop-signals. In line with this, the N2 scalp distribution was generally shifted to the right in addition to an ipsilateral shift in relation to the response hand. Other than that, task lateralization affected neither behavior related to error and feedback processing nor ERN or FRN. Comparing the ERP topographies using the Global Map Dissimilarity index, a large topographic overlap was found between all considered components.With an evenly distributed set of trials and a split-half reliability for all ERP components ≥.85 the task is well suited to efficiently study N2, ERN, and FRN concurrently which might prove useful for group comparisons, especially in clinical populations.

## Introduction

The monitoring and appropriate adjustment of ongoing behavior is essential for adaptive organisms. The anterior midcingulate cortex [1] (aMCC) on the posterior fronto-medial wall, anatomically almost identical to the rostral cingulate zone [2] and also sometimes labeled dorsal anterior cingulate cortex [3], is an important constituent of an executive control network implicated in goal-directed behavior and the possible avoidance of maladjustments. These functions depend on the monitoring of ongoing actions, the processing of performance feedback and the ability to respond or to suppress an initiated response [2]. Some of these processes can be mapped to event-related potentials (ERP) of the electroencephalogram, e.g. the feedback-related negativity (FRN), the error-related negativity (ERN), and the N2 seen in the context of stop-signal tasks.

The FRN follows performance feedback and has its most negative deflection between 200 and 350 ms after feedback presentation. It has a fronto-central scalp distribution and its source has been localized to the aMCC [4], [5], [6], [7]. Initially, the FRN had been associated with the processing of error feedback [5]. More recently, however, it has been interpreted as an indicator of reward prediction and expectancy violations [8], [9], [10], [11].

The error-related negativity (ERN) is an ERP associated with error processing after the commission of an incorrect response in forced choice reaction time tasks, reflecting a mismatch of the executed and intended responses or response conflict monitoring. The ERN is a sharp negative deflection starting at the onset of electromyographic activity preceding the overt erroneous response and peaking about 30 to 100 ms thereafter with a fronto-central maximum and also an assumed source in the aMCC [12], [13], [14].

The ERP components of performance monitoring share some features (e.g. a fronto-central topography and a putative neural generator in the aMCC) but the interrelationship between the components is still unresolved. The relationship between the ERN and FRN was explored earliest [10], [15] with respect to transfer of learning in a probabilistic learning task. Recently the processing of information obtained during a flanker task when the subject realized an erroneous response (internal processing) has been compared to the processing of feedback information given in the same task (external processing) [16], [17]. When the internal information was sufficient, an ERN could be obtained, and only when the external information carried enough additional information did performance feedback elicit an FRN. The authors concluded that ERN and FRN share a functional relationship. The FRN is supposed to rely on the same processes as the ERN, but being expressed only if insufficient internal information is available. Gentsch et al. [16] went a step beyond and used independent component analysis (ICA) to compare the two event-related potentials, suggesting a shared neural network of action outcome updating processes.

With regard to the N2 several variants have been reported in the literature (for a review see: [18]). Here, we report on the negative deflection seen in stop-signal tasks when participants have to withhold a prepared motor response after receiving a stop-signal. It reaches its maximal deflection about 200 ms after the stop-signal and has a larger deflection in trials with successful inhibitions. Like the FRN and ERN the N2 has a fronto-central scalp topography and is also thought to be generated in the aMCC [19], [20], [21]. The N2 might represent the inhibition process or might even index inhibition success [22], [23] but some data suggests that the driving force of the N2 might not be the inhibitory process per se, but predominantly the processing of response conflict. Following this argument, a dissociation between a conflict-driven N2 component and a P3 component more strongly linked to inhibition-related processes has been found [24], [25].

The N2 shares the time-course, morphology and scalp distribution of the aforementioned ERPs. Holroyd et al. [26] compared the FRN elicited in a time-estimation task to the N2 obtained in a separately recorded oddball task and concluded that the FRN is a variant of the N2 and further stipulated that modulations in FRN amplitude result from a positive voltage deflection superpositioned on correct trials. Following this argument Baker and Holroyd [11] demonstrated in a series of carefully designed experiments, that the N2 and the FRN are indeed distinguishable ERP components but may co-occur. In these experiments the N2 was again linked to conflict processing whereas the FRN indexed the processing of rewards. In accordance with the aforementioned notions Yeung and colleagues [27], [28] proposed a common underlying mechanism for the ERN and the N2 in terms of response-conflict, assuming that the monitoring of such conflicts provides a simple way to detect errors as well.

Because the ERN is time-locked to a response it seemed plausible that it is merely affected by response-related processes. However, Dehaene et al. [13] examined erroneous responses committed with either the right or the left hand and did not find conclusive topographical differences. This led the authors to suggest, that the error-monitoring system is independent of exact motor effectors and does rather work on a more abstract level. The assumption is supported by the fact that the ERN is independent of response modality and can be elicited by eye-movement [29], [30], hand or foot responses [31], as well as vocalizations. [32].

Despite the wealth of research on cognitive equivalents of these performance-monitoring ERPs, studies addressing aspects of hemispheric specialization in this domain are rather sparse. An independent functioning of hemispheres as separate cognitive units [33] in contrast to a load-dependent hemispheric division between primary task performance and the implementation of adjustments (e. g. error-correction), has been discussed in the literature [34], [35]. For example, error corrective behavior is impaired when distracting stimuli are presented contralateral to the target [35] suggesting that the task load may mediate the partitioning of task-performance and error-monitoring across the hemispheres. Using two tasks probing known hemispheric specialization (a bargraph judgment and a lexical decision paradigm) and a flanker task with unknown hemispheric preference, eleven participants were tested revealing that across all tasks corrected errors elicited a larger ERN amplitude with right visual field stimulation which was interpreted as predominant processing in the left hemisphere [36]. Unfortunately, however, subjects were asked to give right hand responses only thus compromising hemispheric processing by exclusively invoking the left motor cortex.

In motor-inhibition a network has been implied including the right hemispheric preSMA, the inferior frontal cortex and the subthalamic nucleus in the basal ganglia [37], [38], [39], [40]. Activation in this network has also been linked to reaction-time slowing following stop-signal trials [41]. When comparing ERPs following successful and failed inhibitions in a stop-signal paradigm a right frontal lateralized N2 was obtained in children [42] and young adults [22]. Additionally, higher amplitudes for successful compared to failed inhibitions seemed to index the activation and efficient implementation of the inhibitory process [22]. In accordance with these observations, a recent study using simultaneous EEG/fMRI found that the above described network was associated with both stop- and error-related ERPs in a tactile stop-signal task [43].

Using a Go/Nogo task adapted to fMRI Lütcke and Frahm [44] reported bilateral activations in the aMCC in response to errors (in this case: false alarms, i.e., a motor response after a Nogo cue), whereas correct inhibition was associated with the right aMCC. Hence, the right aMCC was activated for both successful and unsuccessful inhibition, whereas the left aMCC responded solely to false alarms, when the subject committed an error such that its engagement can be attributed to error-processing.

Furthermore, it has been shown that variations in left aMCC morphology are associated with performance differences related to conflict-related processing [45], [46]. Huster et al. [45] found a left-hemispheric dominance (inferred from N2 amplitude differences) in males engaged in a tactile stop-signal task. Using verbal stimuli and visual half-field stimulation with a Go/Nogo task [47] a behavioral advantage of right visual field stimulation compared to left visual field stimulation was observed while the N2 amplitude after Nogo stimuli was attenuated when the stimuli were presented in the right visual field compared to the left visual field. Here, however, the known lateralization of verbal stimuli could have interfered with a thorough analysis of the inhibition process [48], [49].

To our knowledge no attempt to test the functional lateralization of the feedback-driven FRN has been reported yet.

Here, we aim to reliably elicit the three performance monitoring related ERPs (with an aMCC generator, a fronto-central topography and a latency and time-course matching the above given description of the components) in the same session within the same task. We combined the time-estimation task utilized in Holroyd and Krigolson [8] and a stop-signal task and modified them for use in the tactile domain for optimal hemispheric separation. The FRN is to be elicited after the presentation of unexpected feedback. The time-estimation task was designed to include a condition with infrequent – thus unexpected - errors (easy condition) and a second condition with frequent and expected errors (hard condition) allowing us to dissociate the valence of the given performance feedback (correct vs. error) and the expectancy (expected vs. unexpected). Expectations of the participants are violated more by rare errors in the easy condition compared to the frequently occurring errors in the hard condition, and by rare correct responses in the hard condition compared to the more frequent correct responses in the easy condition. Here, in accordance with Holroyd and Krigolson [8], we expect the difference wave isolating effects of unexpected performance feedback to exhibit a more pronounced modulation than the one for expected feedback. The N2 will be elicited in trials with successful inhibitions after the reception of a stop-signal. A failure to inhibit the prepared motor response (i.e. an action slip) will elicit an ERN. The interrelationship between these components will be assessed using bivariate regression analyses.

Recent reports suggest a large overlap and similarity between these performance monitoring ERPs [16], [17], [20] as well as concerning their neural generators [2], [16], [50]. However, according to the abovementioned studies a differential lateralization of these ERPs might be expected, especially with respect to the N2 component. The evidence for a hemispheric processing of error-related signals is much weaker [13], . If at all we expect slightly left hemispheric advantages for the ERN. No differences in hemispheric processing for the FRN are expected. Since we are using non-verbal stimuli we expect to find advantages of the right hemisphere for behavioral indices of inhibitory processing.

## Materials and Methods

### Participants

24 right-handed, healthy young adults (16 female; 26.13±4.64 years old), recruited from the institute's pool of regular participants, participated in the study. All participants volunteered and provided written informed consent. They were paid 15 Euro for participation. The study was conducted in accordance with the ethical standards described in the declaration of Helsinki and was approved by the ethics committee of the University Hospital Münster.

### Apparatus and Procedure

**Figure 1:**
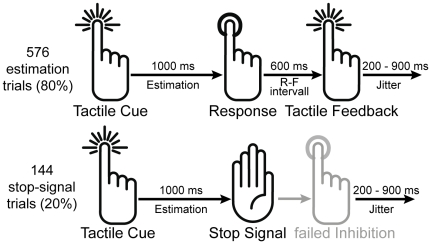
Time-estimation task with interspersed stop-signal trials. The tactile time-estimation task included three conditions (control, easy, hard) with different response-window adaptations leading to different error-rates (see text for details). In the randomly assigned stop-signal trials prior to response execution a stop-signal indicated the need to inhibit the response. Hand symbols from ITT Bombay (www.designofsignage.com).

A task similar to that proposed by Miltner et al. [5] and Holroyd et al. [8], [26] was employed in which participants were required to press a button after they felt one second had elapsed (set up in Presentation v10.3, Neurobehavioral Systems Inc., Albany, U.S.A.). Participants were comfortably seated in a chair placed in a sound attenuated and shielded room. Tactile stimuli were applied by means of a device that translates air pressure, transferred via plastic tubes and acting at a membrane, to tactile stimulations. This stimulation device has already successfully been applied in different studies (e.g. [43]. Clamps hold the membranes attached to a subject's fingertip. Here, these clamps and membranes were used to stimulate the index, middle and ring fingers of the left and the right hands. The strength of the stimulation was adapted as to cause clearly suprathreshold but not painful sensations. Each trial commenced with a tactile cue to the index finger that lasted for 50 ms indicating the beginning of the estimation period. When participants believed that one second had elapsed they were instructed to register this by pressing a button with the index finger. Participants received feedback indicating the accuracy of their estimation 600 ms following the response. A response was considered on time if it occurred within an adaptive response time window (RTW) centered around 1000 ms (see below), and was considered not on time otherwise. Feedback stimuli consisted of tactile cues applied to the middle- or ring finger of either the left or the right hand for a given block of trials. The offset of the feedback was followed by a resting period with a variable duration ranging from 200 to 900 ms (see Figure 1). One block consisted of 30 consecutive trials during which responses were to be given with the very same hand that also received the tactile stimuli. The mapping of positive or negative feedback to the middle or ring finger was counterbalanced across subjects.

The RTW was initialized at 1000 ms±100 ms. If participants responded on time, they received correct feedback in the first trial. In the following trials the size of the RTW decreased, if the response was made within the borders of the RTW and increased otherwise. The amount of this adaptation varied in correspondence with the experimental conditions: *control*, *easy*, and *hard* [8], [26]. In the control condition the window size increased or decreased symmetrically by 10 ms. The RTW grew faster than it narrowed during the easy condition, where it increased by 12 ms on erroneous trials and decreased by 4 ms on correct trials. In the hard condition the window size increased by 4 ms on error trials and decreased by 12 ms on correct trials.

The time-estimation task was interspersed with 20% stop-signal trials. The stop-signal stimulus consisted of a tactile cue to the index finger. The stop-signal was initialized based on the average response time of the last 8 responses minus 150 ms. Following each stop-signal trial, the stop-signal onset asynchrony was decreased by 7 ms following correct inhibition and increased by the same amount after failed inhibitions.

Participants began the experiment by completing two blocks of 30 practice trials of the control condition also interspersed with 20% stop-signal trials. During practice trials visual feedback was provided (correct: green smiley; error: red frowny) in addition to the tactile feedback to accustom participants with the tactile feedback mapping. After this short practice session participants completed eight blocks of the control condition (240 trials in total). The control condition was followed by eight consecutive blocks in each the easy and hard condition, the order of which (control – easy – hard or control – hard – easy) was counterbalanced across participants. Thus, across the three experimental conditions there were 720 trials in total: 576 time-estimation trials, and 144 stop-signal trials. The use of the right- and left hand was consistent in each block but alternated between blocks (every 30 trials).

Participants were informed that some blocks would be more difficult than others, but were not specifically told which blocks were hard or easy. To avoid unnecessary initial adjustments at the beginning of each condition, the RTW established at the end of the control condition served as starting point for both the hard and easy condition. Participants were given self-paced resting periods after every 4^th^ block (120 trials).

### Data Acquisition and ERP parameterization

Response time (in milliseconds) and accuracy (percentage of on time, not on time, and failed inhibition trials) were computed.

**Figure 2:**
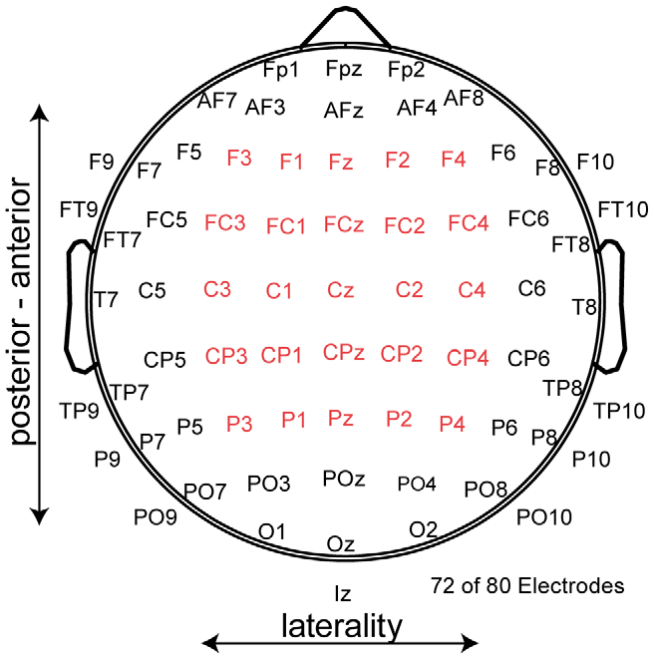
Electrode Array. 72 of the 80 EEG-electrodes used are depicted. The 15 central electrodes were used to compare the peak-amplitudes and topographies of the ERPs. For the Global Map Dissimilarity measure all 80 electrodes were included in the analyses.

Scalp voltage fluctuations were collected using 80 Ag/AgCl scalp electrodes arranged in accordance with the extended 10–20 system (see Figure 2) and recorded using a CTF System (VSM MedTech Ltd., Coquitlam, Canada) with impedances kept under 5 kΩ. The EEG electrodes were referenced online to electrode FCz. Electrodes placed at the infra- and supra-orbital ridges of the right eye monitored vertical eye movements and electrodes placed on the outer canthi of the eyes recorded the horizontal electrooculogram. EEG data were sampled at 600 Hz, and low-pass filtered at 200 Hz.

Offline, the data was filtered between 0.5 Hz and 40 Hz and re-referenced to the common average of all electrodes. After the removal of major artifacts by means of visual inspection and replacement of bad channel data through spherical splines interpolation [51], [52] the continuous datastream was submitted to a temporal extended infomax independent component analysis (ICA) using EEGLAB [53] and custom Matlab 2009b routines (The MathWorks Inc., Natick, U.S.A.). A spatial principal component analysis was used to reduce the dimensionality of the EEG from 80 channels to 30 principal components prior to performing ICA. Independent components representing eye-movement, pulse-, and muscular artifacts were discarded (mean discarded number of components: 9.75±2.5, range 5–16).

To compare the analysis of the FRN to previous reports we followed the steps suggested in Holroyd and Krigolson [8] and created difference waves for each participant: a) one difference wave of the control condition by subtracting the correct ERP from the error ERP, b) a difference wave for the unexpected feedback by subtracting the correct ERP of the hard condition from the error ERP in the easy condition (i.e., infrequent error – infrequent correct), and c) a difference wave for expected feedback by subtracting the correct ERP of the easy condition from the error ERP of the hard condition (i.e., frequent error – frequent correct). The epochs for the FRN spanned from 200 ms prior to 600 ms following the feedback, and were baseline corrected with respect to the 200 ms prior to feedback presentation. The magnitude of the FRN in the described difference waves was defined as the most negative deflection in the 600 ms following the stimulus.

Stop-related epochs (from 200 ms prior to 600 ms post stop stimulus) were extracted and baseline corrected with the mean of the 200 ms pre-stimulus interval. The amplitude of the N2 was then quantified for each participant and channel as the difference between the most negative deflection between 120 ms and 280 ms following the stop-signal and the preceding positive deflection in trials with successful inhibitions.

In order to analyze the ERN, response-related epochs (200 ms pre- to 600 ms post-response) were extracted. The epochs were baseline corrected with the mean of the 200 ms previous to the preceding stop-signal. The amplitude of the ERN was measured for each participant and electrode as the difference between the most negative deflection in the first 120 ms following the erroneous response, failing the signaled inhibition, and the preceding positive deflection. The trough-to-peak measurement utilized here has been suggested as baseline independent quantification for the N2 and ERN [54], [55]. The number of obtained trials for ERP analysis can be seen in Table 1.

**Table 1:** Number of trials for ERP analysis.

	Left Hand	Right Hand
Correct response hard condition	28.3±3.6 (22–35)	26.8±4.8 (19–40)
Incorrect response easy condition	27.0±4.1 (20–35)	27.5±4.6 (17–40)
N2	40.5±3.6 (34–50)	36.4±4.2 (29–44)
ERN	25.7±4.4 (16–32)	28.8±5.4 (17–36)

Mean, standard deviation and range of trials used for ERP analyses. For the analysis of the FRN the difference between the average of the correct responses in the hard condition and the incorrect responses in the easy condition had been calculated.

**Figure 3:**
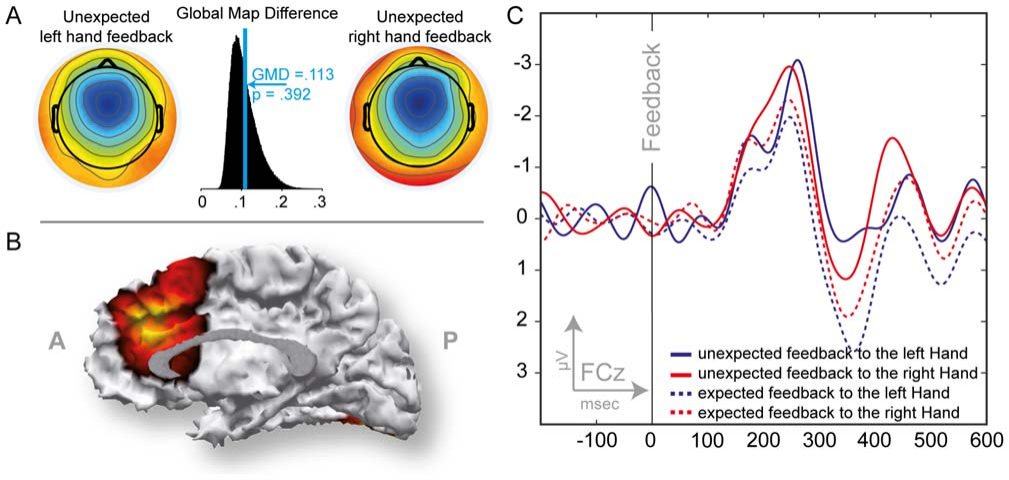
ERP data associated with performance-feedback in the time-estimation task (FRN). A: Scalp topographies for the left and right hand unexpected difference wave FRN peaks and histogram of the Global Map Dissimilarity permutation test between these two topographies. B; SWARM solution for the FRN revealing an aMCC source. C: Difference wave for expected and unexpected outcomes as observed at channel FCz. Tactile performance feedback was received at 0 on the abscissa. Note that negative voltages are plotted upwards by convention.

**Figure 4:**
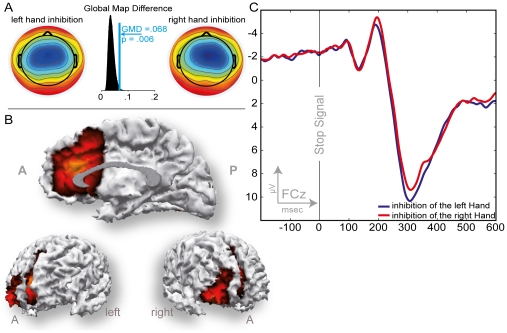
ERP data associated with successful inhibition after a stop-signal cue (N2). A: Scalp topographies for the left and right hand N2 peaks and histogram of the Global Map Dissimilarity permutation test between these two topographies. B: SWARM solution for the N2 revealing the aMCC as well the right inferior frontal cortex as source. C: ERPs recorded at channel FCz. Tactile stop-signal cue given at 0 on the abscissa.

**Figure 5:**
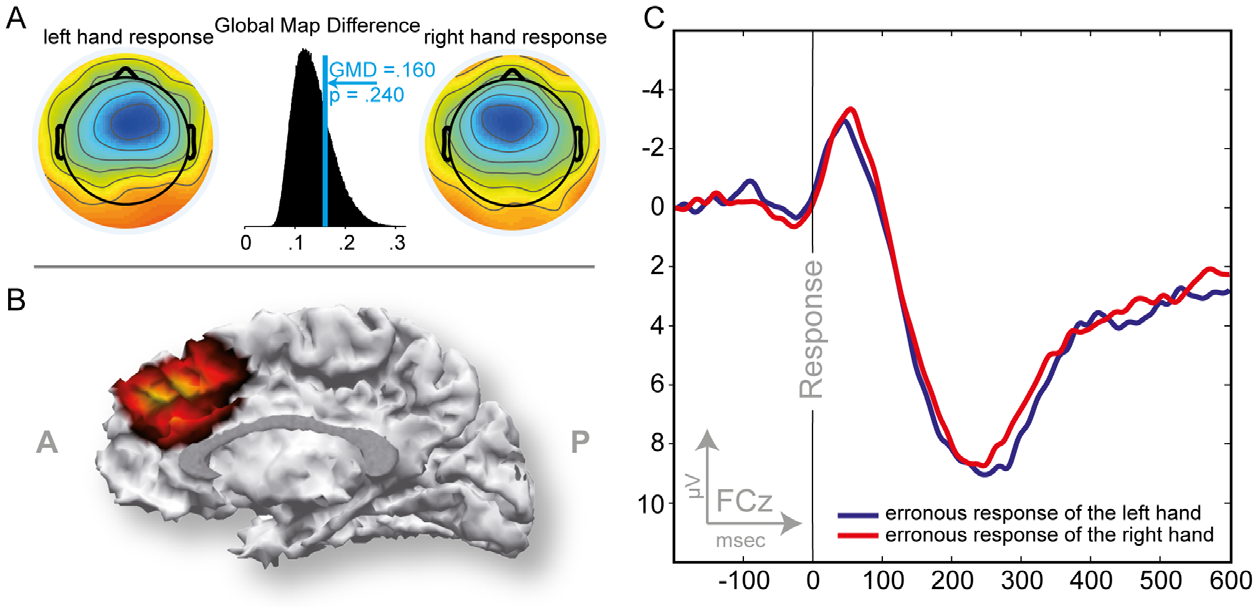
ERP data associated with the action-slip following a stop-signal cue (ERN). A: Scalp topographies for the left and right hand ERN peaks and histogram of the Global Map Dissimilarity permutation test between these two topograhies. B: SWARM solution for the ERN showing the aMCC as source. C: ERPs recorded at channel FCz. Responses were recorded at 0 on the abscissa.

To trace their main generators inverse calculations for the three ERPs of interest (FRN, N2, ERN), averaged across hands and subjects, were computed. Anatomical landmarks (nasion, left and right preauricular points) were used to coregister the electrode positions of one elected subject to its structural MRI. A three-compartment boundary element model was computed for this participant. The resolution of the meshes was set to 9, 8 and 6 mm for skin, skull and brain, respectively. Standard conductivity values for the three compartments were set to: skin = 0.33 S/m, skull = 0.0042 S/m, brain = 0.33 S/m. After gray matter segmentation of the brain, a representation of the cortex excluding the brainstem and cerebellum was computed to limit the source space for the inverse solution. Current density reconstructions (CDR) were calculated using the SWARM method [56], which belongs to the family of weighted minimum norm solutions with its weights being based on a previously computed sLORETA outcome. Figures 3, 4, and 5 exhibit the solutions averaged across data points of 20 ms intervals around the peak alongside the ERPs.

### Statistical Analyses

Erroneous estimations in time-estimation trials, successful inhibitions and errors of commission after stop-signals were calculated as percentages for the whole experiment and for each condition. Differences between CONDITIONS (control, easy, hard), HANDS (left, right), EXPECTANCY (expected, unexpected) and VALENCE (correct, error) of the feedback were calculated using a repeated measure ANOVA. Changes in reaction (estimation) time were calculated as absolute change after erroneous responses compared to response times after correct responses within the RTW. The stop-signal asynchrony was defined as the delay of the stop signal following the estimation cue. A stop-signal reaction time (SSRT) could not be calculated given the characteristics of the underlying time-estimation task. An estimate of a plausible reaction time for each stop-signal trial was derived by calculating the mean of the last 8 estimation trials preceding the stop-signal trial. Possible transfer effects across tasks, especially on the estimation trial following a stop-signal trial will be defined as changes in error rates as well as changes in estimation accuracy in the estimation trial following the stop-signal trial.

ERPs were analyzed by considering the electrodes depicted in Figure 2, covering the midline electrodes Fz, FCz, Cz, CPz, Pz and two-electrode rows lateral to midline. Separate repeated measure ANOVAs were calculated for each ERP with the factors: HAND (left, right), anterior to posterior vector (A-P: Frontal, Fronto-Central, Central, Central-Parietal, Parietal) and LATERALITY (electrode rows left (3 & 1), central, right (2 & 4)).

After the aforementioned ANOVAs revealed electrode FCz as site with the maximal deflection for all considered ERPs we performed the following reliability analyzes: the within-session reliability for the ERPs was calculated by forming separate average ERPs for even and odd trials for the FRN, N2, and ERN. Pearson correlations are reported for the peak measurements of the difference based FRN, and the through-to-peak measurement of the N2 and ERN. Because split-half reliability metrics are based on only half of the trials (odd or even trial number), these measures were corrected using the Spearman and Brown prophecy formula [57]. To assess the interrelationship between the ERPs bivariate regressions were calculated between the FRN and N2, FRN and ERN, and N2 and ERN for the z-normalized peaks (FRN) or through-to-peak (N2, ERN) measurements at electrode FCz.

Lower order effects will only be reported when the nature of higher order effects allow for their interpretation. Greenhouse-Geisser epsilon corrections were computed where appropriate. The software package PASW Statistic 18 (IBM Corporation, New York, USA) was used for statistical evaluations.

In addition to statistical significance testing, effect size estimates were computed using procedures described in [58], [59]. Here, partial eta squared (η_p_^2^) was computed for statistical comparisons. To avoid reporting large amounts of statistical results not relevant to our investigation, only relevant main effects and interactions of post-hoc tests are described.

To compare the whole head scalp topographies the Global Map Differences, as suggested in [60], were calculated (for reviews see: [61], [62]) between the topographies of the potentials elicited through the use of the right or left hand respectively and also between the potentials. In short, this parameter equals the square root of the mean of the squared differences between the normalized potentials measured at each electrode. The resulting Global Map Dissimilarity Index can range from 0 (topographic homogeneity) to 2 (topographic inversion). We used a permutation approach to estimate the probability of our results (sometimes refered to as TANOVA; see [61] for further details). For this approach the single subject maps a) are reassigned to different hand or component condition at a within subject level (permutation of the data), b) the group average ERPs are recalculated, and c) the GMD for this “new” ERP is derived. Based on n participants, in principle 2*^n^* permutations are possible but it has been suggested, that about 1,000–5,000 permutations are sufficient [63]. Here we used 100,000 permutations for all tests.

## Results

### Behavioral Data

#### Time-estimation

**Figure 6:**
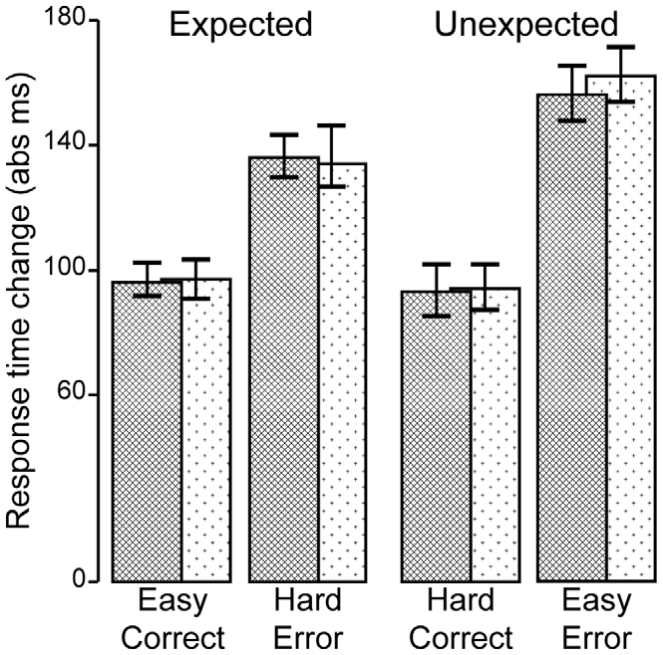
Performance data in the time-estimation task. Absolute changes in response time following expected (correct easy trials and erroneous hard trials) and unexpected outcomes (correct hard trials and erroneous easy trials).

The behavioral data indicate a successful manipulation of time-estimation success (*F*_2,46_ = 2083.48, *p*<.001, η_p_^2^ = .99): in the control condition participants were correct on about ½ of the trials (left hand (LH): 48.5%; right hand (RH): 50.2%), in the hard condition in about ⅓ of the trials (LH: 29.9%; RH: 28.3%), and in ⅔ of the easy trials (LH: 71.8%; RH: 70.9%). No differences between LH and RH stimulations were seen (*F*<1). Consistent with the error rate, the median size of the response window was smaller in the hard condition (LH: 112 ms ±39; RH: 108 ms ±39) than in the easy condition (LH: 316 ms ±89; RH: 321 ms ±103)( *F*_1,23_ = 206.50, *p*<.001, η_p_^2^ = .90). Again no effect of hand or interaction of hand and condition were observed. The absolute change in reaction time was larger on trials that immediately followed error trials than on trials that immediately followed correct trials in all conditions (*F*_2,23_ = 268.36, *p*<.001, η_p_^2^ = .92), with the smallest adaptation after correct responses in the hard condition (*F*_2,46_ = 3.95, *p* = .026, η_p_^2^ = .15)(see Figure 6).

Since we were also interested in putative effects due to differences of the valence of the feedback or the expectancy, we subjected the absolute change in response time on the following trial to a repeated measure ANOVA including the factors hand, expectancy and valence. This revealed a main effect of valence (*F*_1,23_ = 161.69, *p*<.001, η_p_^2^ = .88), a main effect of expectancy (*F*_1,23_ = 8.99, *p*<.006, η_p_^2^ = .28), and an interaction between expectancy and valence (*F*_1,23_ = 10.86, *p* = .003, η_p_^2^ = .32) leading to greater change in reaction time especially following unexpected error feedback. No main effect of or interaction with hand was observed (*F*<1).

#### Stop-signal trials

In about half of the stop-trials participants successfully inhibited their responses. Interestingly, the percentage of successful stops-trials was larger with left- as compared to right-hand stimulations (LH: 56.6% ±5.1; RH: 50.7% ±5.1; *F*_1,23_ = 9.17, *p* = .006, η_p_^2^ = .29). An approximation of the SSRT, the distance between the stop-signal and the mean reaction time of the preceding eight reactions, was 192 ms ±26 for the left hand and 193 ms ±28 for the right hand (no significant difference, *F*<1). Possible conflicts in information processing, which could arise from inhibitory mechanisms, did not seem to spread to the following estimation trial: neither did error rates nor the estimation accuracy after stop-signal trials change between the estimation-trials preceding the stop-signal trial and the estimation-trial following the stop-signal trial (*F*<1).

No behavioral differences in inhibition performance or erroneously committed responses after stop signals were found between the three difficulty conditions of the time-estimation task. During the control condition the stop signal was presented earlier in comparison to both other stages (Control: 1212 ms ±50, Easy: 1162 ms ±67, Hard: 1161 ms ±79; *F*_1.68,38.59_ = 6.65, *p* = .005, η_p_^2^ = .22) with no differences between the easy and hard conditions. This might be due to a learning effect and consequent adjustment of inhibition performance since the control condition was always presented first.

### Electrophysiological Data

#### FRN

Difference waves for expected and unexpected outcomes and for the control condition exhibited a fronto-central scalp distribution with a maximal deflection at electrode FCz without significant differences due to hand.

The peak amplitude of the difference wave for unexpected outcomes was larger than the peak amplitude of the difference wave for expected outcomes (Table 2 & Table 3) yielding a significant difference between conditions but not between hands (*F*_1,23_ = 16.90; *p*<.001; η_p_^2^ = .424, and *p*>.2 for the factor hand). The scalp topography for unexpected outcomes appeared wider and more frontal as compared to the scalp topography for expected outcomes (Figure 3). No effect of hand, used to receive feedback, was observed in either condition.

**Table 2:** ERP amplitudes at electrode FCz.

	Left Hand	Right Hand
FRN expected feedback	−3.78 µV±2.46	−4.05 µV±2.01
FRN unexpected feedback	−5.62 µV±2.98	−6.13 µV±3.44
N2	−6.14 µV±2.94	−6.81 µV±3.94
ERN	−5.69 µV±2.97	−5.77 µV±2.82

**Table 3:** FRN-related ANOVA with factors Expectancy (Expected, Unexpected), Hand (left, right), A-P, and Laterality.

Factor	df	F	p	η_p_^2^
Expectancy	1	23.18	<.001	.502
Laterality	1.86	12.62	<.001	.354
Expectancy * AP	1.55	4.00	.037	.148
A-P* Laterality	4.23	4.59	.002	.166

The GMD index for comparing the difference waves for receiving unexpected feedback on the left and right hand did not reveal differences of the scalp distribution (*GMD* = .113, *p*>.1). Source localization suggests a generator in the posterior medial frontal wall including aMCC (Figure 3B).

#### N2

Following the stop-signals with a successful response inhibition, a negative deflection was present in the ERPs. The peak amplitude across all conditions of the time-estimation task was maximal at FCz. No influences of the conditions' difficulty of the time-estimation task on the N2 component regarding its peak amplitude or topography were observed. Through all conditions a fronto-central scalp distribution was present with a central-right maximum for both hands. The topography was shifted to the ipsilateral side of the stop-stimulus receiving hand as indicated by the significant interaction of HAND and topographical factors (Table 2 & Table 4) and the GMD permutation (*GMD* = .068, *p* = .006). The main effect of LATERALITY confirms the visual impression of a general rightward shift of the N2 independent of response hand (Figure 4). Source localization suggests generators in the posterior medial frontal wall including aMCC and in the right inferior frontal cortex (Figure 4B).

**Table 4:** N2-related ANOVA with factors Hand, A-P, and Laterality.

Factor	df	*F*	*p*	η_p_^2^
AP	1.72	4.25	.02	.156
Laterality	2.10	2.88	.06	.111
Hand * Laterality	1.88	19.99	<.001	.465
AP * Laterality	3.95	2.73	.034	.106
Hand * AP * Laterality	4.37	4.82	.001	.173

#### ERN

Errors of commission after the stop-signal elicited an ERN that reached its maximum at electrode FCz (Figure 5). The amplitude peaked about 60 ms after the response with no significant differences between hands or conditions of the time-estimation task (Table 2 & Table 5).

**Table 5:** ERN-related ANOVA with factors Hand, A-P, and Laterality.

Factor	df	*F*	*p*	η_p_^2^
AP	1.78	12.81	<.001	.358
Laterality	1.85	12.15	.001	.346
AP * Laterality	5.11	2.49	.034	.098
Hand * AP * Laterality	4.50	3.43	.009	.130

Overall, the scalp distribution of the ERN was fronto-central and leaned to the hemisphere contralateral to the response hand within the electrode array analyzed with the ANOVA. However, this topography shift did not reach significance, which is also supported by the GMD permutation comparing left and right hand errors (*GMD* = .160, *p*>.1). Source localization suggests a generator in posterior medial frontal cortex including aMCC (Figure 5B).

### Comparison and Reliability

For the N2 the ANOVA and the GMD indices suggested a right lateralized topography with successful inhibition of the right hand, and a more central topography with successful inhibitions of the left hand. The ERN and the FRN showed no lateralization.

Utilizing the GMD measure we also compared the normalized scalp distribution of the same hand between all components. This analysis revealed GMD distribution means below 0.15 (see Table 6). Considering the range of the GMD (0–2) this suggests highly similar scalp topographies with only minor differing constituents.

**Table 6:** Global Map Dissimilarity scores comparing the ERPs.

Comparison	left hand		right hand	
	*GMDscore*	*p*	*GMDscore*	*p*
FRN vs. N2	.21	.001	.14	.007
FRN vs. ERN	.19	.211	.24	.007
N2 vs. ERN	.26	<.001	.29	<.001

Using 100,000 permutations the Global Map Dissimilarity between two ERP topographies is estimated. The GMD-score ranges from 0 = identical topographies to 2 = inverted topographies.

**Figure 7:**
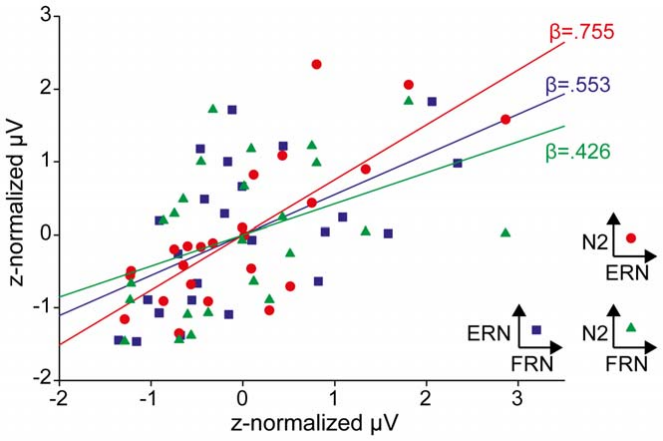
Regression analyses between FRN, N2, and ERN. Peak amplitudes of the ERPs were z-normalized. Bivariate regression analyses between the N2 and ERN revealed a strong relationship (*β* = .755; SEM = .140; *t* = 5.407; *p*<.001) between the N2 and ERN, and much less shared variance between the N2 and FRN (*β* = .426; SEM = .193; *t* = 2.21; *p* = .038). Coefficents of the bivariate regression between ERN and FRN are in between the aforementioned ones (*β* = .553; SEM = .178; *t* = 3.11; *p* = .005).

Bivariate regression analyses between the z-normalized peak amplitudes of the three components revealed strong associations between the FRN, N2, and ERN amplitudes. The relationship between the amplitudes of the N2 and ERN was strongest (*β* = .755; SEM = .140; *t* = 5.407; *p*>.001; R^2^ = .571), whereas the relationship between the N2 and FRN was much weaker (*β* = .426; SEM = .193; *t* = 2.21; *p* = .038; R^2^ = .181). The relationship between ERN and FRN amplitudes was in between (*β* = .553; SEM = .178; *t* = 3.111; *p* = .005; R^2^ = .306). These results are also depicted in Figure 7.

For all ERPs the calculated split-half reliability was high (FRN: r = .85, *p*<.001; N2: r = .91, *p*<.001; ERN: r = .89, *p*<.001) indicating that with the task at hand the ERPs of interest could be elicited and measured reliably.

## Discussion

In the present study we designed a task to elicit three event-related potentials known to reflect performance-monitoring processes: the feedback-driven FRN, the stop-related N2, and the ERN following an action slip. To this end we used a completely lateralized, tactile time-estimation task interspersed with infrequent stop-signal trials while recording EEG. We aimed to reliably measure these ERPs, associated scalp topographies and also to discern potentially different laterality patterns or hemispheric processing preferences of these components.

The expectancy manipulation proposed in [8] with an asymmetrical adjustment of the response window for time-estimations after correct and erroneous estimations lead to error rates for the easy and hard condition of 1/3 and 2/3, respectively. Hence, in the easy condition the participants expected to have estimated the time interval appropriately in the majority of trials, whereas in the hard condition an erroneous estimation was expected to occur more often. In about half of the interspersed stop-signal trials the participants were able to successfully inhibit their prepared motor-response, and failed in the remaining trials.

### Lateralization

Hemispheric differences were found only for the stopping condition and the associated *N2*. Participants were more successful in inhibiting prepared responses of the left hand as compared to the right hand, suggesting a right hemispheric inhibition advantage. Whereas N2 peak amplitudes were not affected by lateralization of stimuli and responses, a complex topographical shift was observed: The generally fronto-central N2 scalp distribution was shifted ipsilateral to the stimulation/response side, but more so for right-sided than for left-sided stop trials. The latter finding suggests a general right-shift in the topography, which is supported by source localization in which in addition to the aMCC the right inferior frontal cortex seems to contribute to N2 generation. Both, the topographical right shift and the right inferior frontal source had been expected based on previous studies reporting right lateralized N2 topographies [22], [42]. Also fMRI and lesions studies describe a right hemispheric network as key element in response inhibition [37], [38], [41] as well as in conflict monitoring [44]. Our findings contrast a prior finding where at least male subjects showed a pattern suggestive of left-hemispheric dominance in N2-related processes [45] with a larger N2 amplitudes evoked by right-hand stimulations. An explorative analysis of our data, calculated separately according to sex, did not reveal any behavioral differences or different ERP amplitudes between the sexes. Given that leftward MCC folding asymmetries, signifying a larger aMCC in the left hemisphere, has been shown to be associated with increased performance monitoring capabilities and ERPs [46], [64], [65], this issue needs further investigation.

The additional ipsilateral topographical shift of the N2 depending on stimulus/response side might be explained by several accounts. Recently, functional suppression of activity in the contralateral motor cortex during motor inhibition had been suggested [40], [66]. According to this notion, an already prepared motor-plan has to be suppressed and this inhibition process will lead to a relative increase of ipsilateral motor cortex activation. The ipsilateral shift of N2 topographies observed in with our task might lend further support to this idea. However, the N2 does not overlap with beta band activity in the motor cortex which has most consistently been associated with motor inhibition [67]. Alternatively, if the N2 is associated with inhibitory activity or response conflict on stop trials, its medial frontal source might be dependent on the side of the prepared motor response or input of the stop signal.

An *ERN* topography most pronounced contralateral to the response hand had been suggested [68] and attributed to motor processes and an inhibition of the ipsilateral motor cortex through synchronized theta-band oscillations. However, neither advantages in favor of left-hemispheric processing, nor differences in peak amplitude which were to be expected with right-handed participants [36], nor a shift in topographies for the ERN was observed in this study. Similarly, the *FRN* did not show any topographical shifts or amplitude modulations dependent on response/feedback hand. This seems to suggest that the processes underlying ERN and FRN do not exhibit a strong hemispheric asymmetry. For the N2, this seems to be different, suggesting that it (a) at least partly does reflect different processes, (b) is associated with the motor system and/or (c) shows a higher degree of hemisphericity than the other two components. For all three ERPs a source in the aMCC was obtained and only minor topographical differences were present, suggesting a large functional overlap. As suggested in [16], [17] the FRN and ERN reflect closely related processes and are likely to rely on a shared network for updating action outcome processes. The topographic differences found between the components may also reflect the differential contribution of simultaneously ongoing processes linked to either the motor response, motor inhibition or sensory (feedback) input, without being directly associated with performance monitoring per se. Pair-wise regression analyses also indicated strong similarities between all three ERPs, suggesting that the ERN shares significant commonalities with the other two performance monitoring ERPs. However, it should be noted that the current study, while suggesting many commonalities between the processes reflected by N2, ERN and FRN, cannot test or integrate theories of performance monitoring that, up to now, each can link only two of the components (e.g., conflict monitoring: N2, ERN [20], [69]; mismatch and reinforcement learning theories: ERN, FRN [10]).

### An efficient paradigm for reliable tests of performance monitoring

Our results suggest that the presented new paradigm combining adaptive time estimation and a stop signal task is well suited for efficiently eliciting three robust and reliable ERP correlates of performance monitoring. The two-fold task-adaptations (a staircase procedure for the stop-signal delay and the adaptation of the response time window for the time-estimation) led to an evenly distributed number of trials for optimal analyzes of the ERPs, avoiding frequency effects. We obtained high reliability indices for all ERPs (>.85) establishing the task as a reliable tool to research group differences. Only for the ERN similarly high values for split-half reliabilities for a peak measurement were reported before [70]. The reliability of the FRN has not been investigated before. High reliability indices are especially important when these components or specific differences between these components are used in clinical settings to establish groups or endophenotypes, as has been suggested for obsessive-compulsive disorder [71]. No specific value of reliability can be considered a cutoff for acceptability, but Helmstadter [72] suggested .5 for group studies and in clinical relevant individual assessments .94, so that ERPs are rarely used for individual clinical diagnostic assessments but still prove highly useful for research.

When lateralization is not of interest, the same paradigm could easily be transferred to the visual domain, and, given the high reliability of the ERPs, the trial number could be reduced, such that the duration would become less than 30 min. Indeed, in one of our pre-studies, conducted to confirm the behavioral adaptation effects of the task, we already used visual cues with success.

As such, this novel paradigm presented here might be a useful tool for the study of inter-individual differences or pathological changes in clinical populations [73]. Considering the different sources of information processed for effective performance-monitoring with this paradigm (e.g. internal error monitoring vs. external task- and feedback-stimuli) and cognitive processes targeted with this task, it is possible to disentangle whether pathology affects the performance monitoring system as a whole [2] or rather its subcomponents.

Patients with Tourette's Syndrome might serve as an example of a clinically relevant population as augmented inhibitory control in patients has been postulated [74], [75]. Increased inhibitory control has been associated with an activation of prefrontal brain regions during tic suppression [76] and was found during performance of a demanding cognitive task [77]. On the other hand, performance-monitoring per se or error-processing behavior was not affected [78]. Similarly, in obsessive-compulsive disorder (OCD) hyperactive error processing has consistently been found (e.g. [79], [80], [81]), but possible impairments in context of expectancy violations or inhibition have not yet been explored systematically. Beyond this, it was suggested that with this population the processing of internal performance measures in probabilistic learning tasks stands in contrast to internal performance measures in simple choice reaction time tasks [82]. Impulse control, especially inhibitory control, is also studied in the context of attention-deficit/hyperactivity disorder where the N2 amplitude is reduced in ADHD children [42]. A reduced ERN amplitude has also been found in an ADHD group suggesting a global impairment in cognitive control [83] which could now be tested using one task in one single short session (∼30 min).

In conclusion our paradigm is a reliable instrument to elicit the FRN, N2 and ERN within one task. Furthermore, our data support a common adaptation network associated with the aMCC. A hemispheric preference and shift in related scalp topographies was only observed for the N2. Neither for the FRN nor ERN this observation could be made.
